# [2+2]-Photocycloadditions of 1,4-Naphthoquinone Under Batch and Continuous-Flow Conditions

**DOI:** 10.3390/molecules29245920

**Published:** 2024-12-15

**Authors:** Madyan A. Yaseen, Zhifang Guo, Peter C. Junk, Michael Oelgemöller

**Affiliations:** 1 College of Science and Engineering, James Cook University, Townsville, QLD 4811, Australia; madyan.yaseen@uosamarra.edu.iq (M.A.Y.);; 2 College of Education, University of Samarra, Samarra 34010, Salah Al-Deen, Iraq; 3 Faculty of Chemistry and Biology, Hochschule Fresenius gGmbH—University of Applied Science, 65510 Idstein, Germany

**Keywords:** photocycloaddition, naphthoquinones, cyclobutanes, continuous-flow photochemistry

## Abstract

A series of [2+2]-photocycloadditions of 1,4-naphthoquinone with various alkenes and diphenylacetylene were investigated under batch and continuous-flow conditions. Acetone-sensitization furnished the corresponding photoadducts in good to excellent yields and purities. Compared to batch operations that demanded exhaustive irradiation times of 10–13 h, the flow process generally gave superior conversions and subsequently yields with a residence time of just 60 min. The structures of several photoaddition products were further determined by crystal structure analysis.

## 1. Introduction

**Scheme 1:**
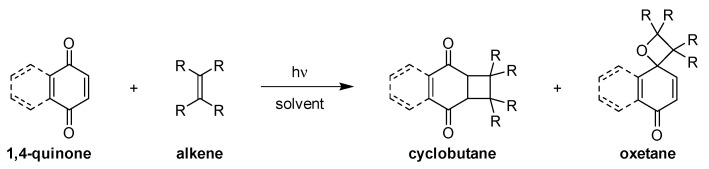
[2+2]-Photocycloadditions of 1,4-quinones with alkenes.

Due to their importance as natural products with a broad range of biological activities, quinones have attracted considerable attention from the chemical and biological communities [1,2,3,4]. Of the many photochemical transformations of quinones [5,6,7], [2+2]-photocycloadditions with alkenes, cycloalkenes and alkynes have been especially intensively studied. Depending on the substrates and irradiation conditions, the photoaddition of alkenes delivers cyclobutanes and spiro-oxetanes (Scheme 1), respectively [8,9,10,11]. These photoadducts have also been investigated for their biological activities [12,13].

Most experimental procedures are conducted in conventional batch reactors using hazardous solvents, such as benzene or acetonitrile, and harsh light conditions, such as medium-pressure mercury lamps [14,15]. The latter commonly cause decomposition of the photoproducts due to over-irradiation, necessitating optical filters that significantly reduce the energy efficiency of the lamps. Recently, continuous-flow chemistry has revolutionized chemical process technologies, especially for on-site and on-demand manufacturing of pharmaceuticals or other commodity chemicals [16,17,18]. The naturally small inner dimensions and the flow operation are especially advantageous for photochemical operations [19,20,21,22]. In particular, light can efficiently penetrate the thin reaction channels, and photoactive products are rapidly removed from the illuminated area. At the same time, new light sources enable more precise excitation of photoactive compounds [23]. Despite these recent developments in photochemical science and engineering, photochemical reactions involving quinones have only been sparsely investigated under continuous-flow conditions [24,25]. To fill this gap, this study investigated photocycloadditions of 1,4-naphthoquinone with a series of alkenes and diphenylacetylene under batch and flow conditions and compared their performances.

## 2. Results and Discussion

### 2.1. Photocycloadditions Under Batch Conditions

For irradiations under batch conditions, a modified procedure described by Maruyama et al. was selected [26]. Irradiations were conducted using Schlenk flasks in a Rayonet photochemical chamber reactor equipped with sixteen 8 W lamps (outside-in irradiation). A five-fold excess of alkene was employed to suppress undesired photodimerizations of the quinone [27]. The reaction mixtures were degassed and purged with a stream of N_2_ during operation. All photoreactions were monitored by TLC or by ^1^H-NMR spectroscopy.

#### 2.1.1. Optimization Studies

**Scheme 2:**
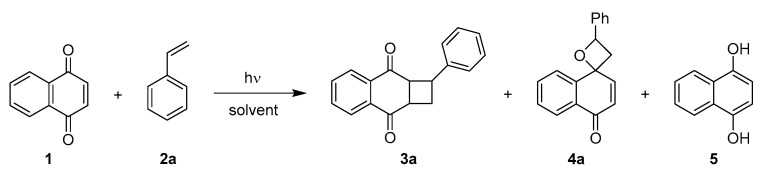
[2+2]-Photocycloaddition of 1,4-naphthoquinone with styrene as a model reaction.

The photocycloaddition of 1,4-naphthoquinone (**1**) with styrene (**2a**) to the corresponding cycloadducts **3a** and **4a** was initially used as a model system to investigate the reaction conditions (Scheme 2). A number of parameters, i.e., the choice of solvent, wavelength, glass type and light source were examined.

##### Solvent Optimization

To find the best reaction medium, degassed solutions of 1,4-naphthoquinone (**1**, 1 mmol) and styrene (**2a**, 5 mmol) in different organic solvents (50 mL) were irradiated with UVB light (300 ± 25 nm) in a Pyrex Schlenk flask (transmission >300 nm) for 10 h. The experimental results are summarized in Table 1.

The photocycloaddition showed two main reaction pathways: cyclobutane (**3a**) or oxetane (**4a**) formation, and the dominant chemoselectivity depended on the solvent applied. The energy levels of the (n,π*) and (π,π*) excited states of 1,4-naphthoquinone are very closely spaced but are sensitive to solvent polarities [8,28]. Photoreactions by direct excitation of **1** in acetonitrile, trifluorotoluene or chloroform produced mixtures of the cyclobutane **3a** and the spiro-oxetane **4a**, of which **3a** could be isolated in pure form in yields of 51–58% (entries 1–3). Under these conditions, both excited states of **1** are populated simultaneously. The observed deviations in chemoselectivities can be attributed to the sensitivity of the excited states and of the reaction intermediates involved to solvent polarities [8,28,29]. In contrast, triplet sensitization by acetone (T_1_ = 332 kJ/mol vs. **1**: T_1_ = 241 kJ/mol [30]) solely yielded 69% of the cyclobutane adduct **3a** with no trace of **4a** detectable in the crude reaction mixture (entry 4). This outcome suggests that triplet excited acetone selectively populates the π,π* triplet state of 1,4-naphthoquinone or that the polar nature of this solvent significantly lowers the π,π* triplet state energy of **1**. Acetone also had practical advantages, as it was easily removed by evaporation, kept all reagents and products dissolved and prevented photodegradation of the cyclobutane photoproduct **3a**. The photoreaction in methanol underwent rapid photoreduction to the corresponding naphthohydroquinone **5** instead (entry 5) [31].

The photoprotective nature of acetone was further demonstrated in a photostability study. Solutions of compound **3a** in either acetone or acetonitrile (T_10%_ = 190 nm [30]) were exposed to Pyrex-filtered UVB light for 8 h. No reaction was observed in acetone, and **3a** was recovered nearly quantitatively. In contrast, irradiation in acetonitrile initiated significant decompositions due to the possible excitation of **3a** (λ ≤ 315 nm) in this solvent. ^1^H-NMR analysis of the recovered material suggested the formation of an aldehyde, possibly through Norrish type I cleavage [32]. No attempts were made to isolate any of the degradants.

##### Wavelength Optimization

The absorption spectrum of 1,4-naphthoquinone shows λ_max_ at 246, 330 and 425 nm in hexane, with the first two due to π,π* and the last due to n,π* transitions [33]. These transitions can be utilized through selection of the lamp emission and the glass type of the reaction vessel. Thus, a series of photocycloadditions of the **1**/**2a** model pair in a Pyrex (λ ≥ 300 nm [30]) or quartz (λ ≥ 200 nm [30]) vessel were conducted with different light tubes and in various organic solvents. The results of the irradiation experiments are summarized in Table 2.

Independent of the solvent, irradiations with visible light produced regioisomeric mixtures of **3a** and **4a** (entries 1–4). Under these conditions, both excited states of 1,4-naphthoquinone are populated through direct excitation (**1**: λ ≤ 460 nm [6,7]). When UV light and acetone were used, high conversion rates and selectivity were instead observed (entries 5–8). With its absorption beginning in the UVA range (T_10%_ = 329 nm [30]), acetone is predominantly excited and operates as a triplet-sensitizer [28]. Irradiation with UVC light in acetonitrile solely yielded cyclobutane (**3a**) by direct excitation of **1** to its π,π* triplet state (entry 9). As **3a** and styrene (**2a**) show absorption bands at 254 and 248 nm themselves [6,7,8,9], competing light absorption by these compounds slowed the photocycloaddition. The formation of polymeric by-products from styrene was further confirmed by ^1^H-NMR analysis of the crude product. Based on these results, the combination of acetone as a solvent and Pyrex-filtered light emitted from a UVB lamp was identified as the ideal irradiation conditions for preparative photocycloadditions (entry 6).

#### 2.1.2. Photocycloadditions with Other Alkenes

**Scheme 3:**
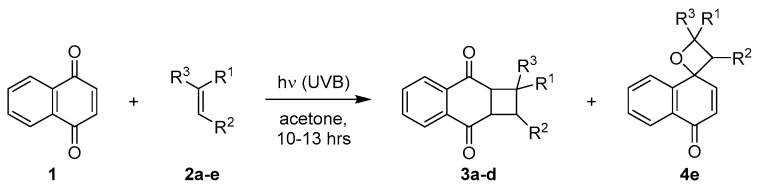
[2+2]-Photocycloadditions of 1,4-naphthoquinone with various alkenes.

The optimized reaction protocol was subsequently transferred to other alkenes and cycloalkenes (Scheme 3). Prolonged irradiations for 10–13 h furnished conversion rates of 40–96%, as determined by ^1^H-NMR analyses of the crude reaction mixtures. The photoproducts, either the cyclobutanes **3a–d** or the oxetane **4e**, were isolated by automated chromatography in acceptable to good yields of 25–78% (Table 3). The isolation process naturally caused losses of material [34].

##### Photocycloaddition with Styrene

**Figure 1:**
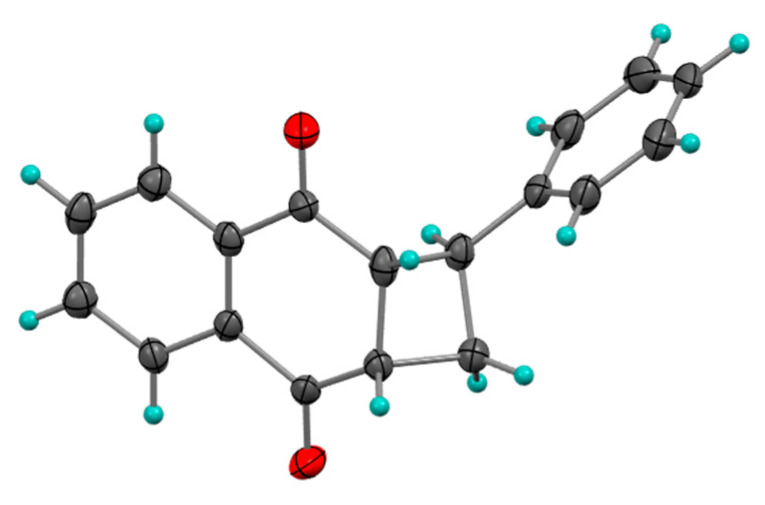
Crystal structure of *anti*-**3a** (from methanol).

The photoreaction with styrene **2a** for 10 h resulted in a conversion of 96% and furnished an approx. 3:1 mixture of the *anti*:*syn* isomers of **3a** in a combined yield of 69% (entry 1). The presence of the two isomeric products was evident from two complete sets of ^1^H-NMR signals in the crude product. The characteristic signals to determine the isomeric ratio appeared at 3.46 ppm for the major *anti*-stereoisomer, whereas the minor *syn*-isomer occurred in the range of 3.63–3.69 ppm. Bryce-Smith et al. reported a similar reaction in benzene using a medium-pressure mercury arc lamp and obtained cyclobutane **3a** in 40% with a stereoisomeric ratio of 2:1 instead [35]. The *anti*-isomer was subsequently crystallized from methanol while the *syn*-isomer was precipitated with trifluorotoluene. The structures of the separated isomers were unambiguously confirmed by NMR spectroscopy in comparison with the literature data [35]. The preference for the *anti*-isomer can be attributed to the preferred *trans*-configuration of the phenyl group with respect to the dihydronaphthoquinone ring system. The structure of the major *anti*-isomer was, furthermore, established by X-ray crystallography (Figure 1). The conformation of the cyclobutane was puckered, with bond angles between 87–89°. The five hydrogen atoms along the cyclobutane ring were not quite eclipsed, whereas the phenyl substituent occupied a pseudo-equatorial position [36].

##### Photocycloaddition with 1,1-Diphenyl Ethylene

**Figure 2:**
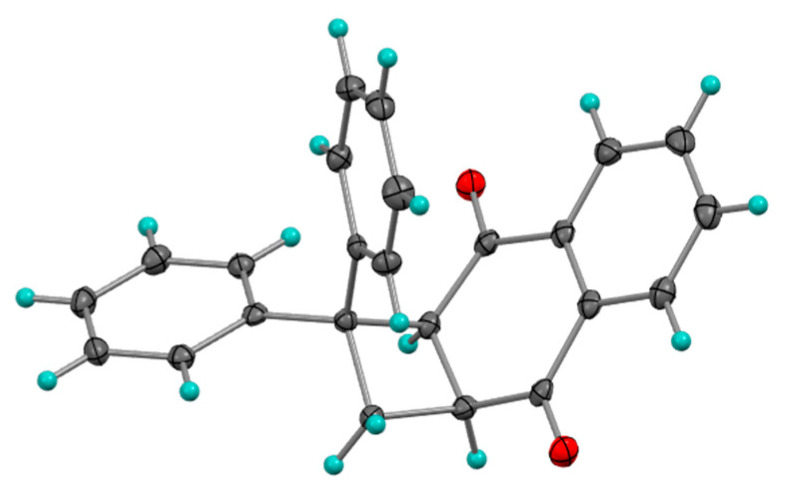
Crystal structure of **3b** (from methanol).

The photocycloaddition of 1,4-naphthoquinone (**1**) with 1,1-diphenyl ethylene (**2b**) required 12 h to reach a conversion of 78% and yielded the corresponding cyclobutane **3b** in a yield of 42% (entry 2). The structure of the photoproduct **3b** was confirmed by X-ray crystallography (Figure 2). The cyclobutane ring possessed a puckered structure with a dihedral angle of *ca* 15° [36]. Four hydrogen atoms and one phenyl substituent were placed in pseudo-equatorial positions, while the second phenyl ring occupied a pseudo-axial position. The bond angles were found to be between 87–90° and the hydrogen atoms along the cyclobutane ring were not completely eclipsed. Overall, the data obtained from the X-ray analysis corresponded well with the recorded NMR data.

##### Photocycloaddition with Cyclopentene

**Figure 3:**
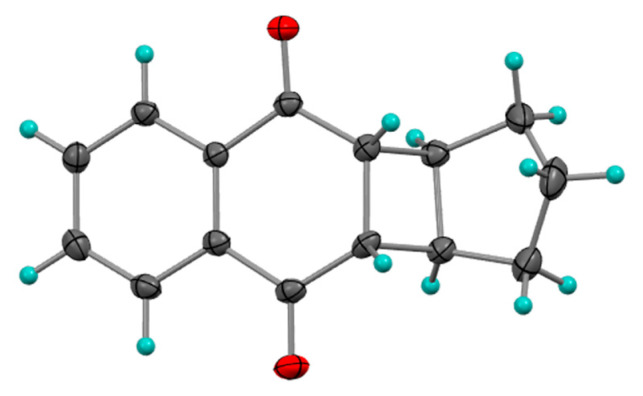
Crystal structure of *anti*-**3c** (from *n*-hexane/ethyl acetate).

Irradiation of **1** with cyclopentene (**2c**) for 13 h produced an incomplete conversion of 67% and solely yielded the *anti*-cyclobutane **3c** in 50% after purification (entry 3). The complete diastereoselectivity may be explained by the increased steric bulk and flexibility of the cyclopentane moiety in **3c**. The ^1^H-NMR spectrum of *anti-***3c** in CDCl_3_ showed a pair of doublets at 2.90 ppm and 3.08 ppm for the protons on the cyclobutane ring with ^3^*J*_cis_ couplings of 4.4 Hz and 3.3 Hz, respectively. The photoreaction was previously investigated in benzene by Bryce-Smith et al. and produced the same isomer, *anti-***3c**, in a much lower yield of just 10% after several hours of irradiation [35]. In contrast, Maruyama et al. reported that no cycloaddition reaction was observed upon irradiation in a benzene solution. Instead, photoreduction to **5** via hydrogen abstraction from the allylic position of cyclopentene was noted [26]. This somewhat contradictory finding may again be explained by different excited state pathways with photoreduction operating via the n,π* and acetone-sensitization via the π,π* triplet state, respectively. The crystal structure of *anti-***3c** is shown in Figure 3. The cyclobutane adopted a much shallower conformation, with all bond angles >89°. The *cis* protons did not appear to be completely eclipsed, with dihedral angles of 6–8°. In contrast, the bond angles in a typical puckered structure have been determined to be 88° and the dihedral angles to be 28° [36,37]. The cyclopentane ring adopted an envelope conformation, with its ring protons in a skew conformation. The C–C–C bond angles varied from 104–106°, resulting in an asymmetrical envelope conformation.

##### Photocycloaddition with Cyclohexene

**Figure 4:**
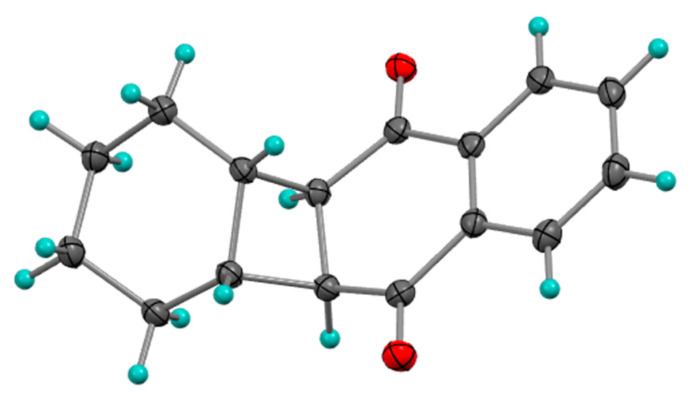
Crystal structure of *anti*-**3d** (from ethanol).

After 12 h of irradiation, the reaction of 1,4-naphthoquinone (**1**) with cyclohexene (**2d**) reached a conversion of 84% and furnished the cyclobutane adduct *anti*-**3d** in a low yield of 25% due to exhaustive purification (entry 4). In comparison, irradiation in a benzene solution only produced photoreduction to **5** instead [26]. The ^1^H-NMR spectrum of **3d** revealed the two *cis* protons as narrowly spaced doublet of doublets at 3.35 ppm. In the corresponding ^13^C-NMR spectrum, the two ethylene bridges of the cyclohexane ring gave two peaks, at 22.3 and 27.5 ppm, respectively. The crystal structure of the photoadduct *anti*-**3d** is furthermore shown in Figure 4. The cyclobutane ring is somewhat less puckered, with a dihedral angle of 22°, whereas the cyclohexane ring adopts a near chair conformation [36]. In the cyclobutane ring, two types of C–H bonds were identified: pseudoaxial and pseudoequatorial. The puckered conformation of the cyclobutane exhibited bond angles of 87–88°. The constraint imposed by the *anti*-fusion of the rings forced the cyclohexane ring to adopt a slightly distorted and flattened chair conformation. As a result, the bond angles were found in the range of 111–120°.

##### Photocycloaddition with Trans-Stilbene

The photoreaction of **1** with *trans*-stilbene (**2e**) is known to produce solely the spiro-oxetane via an electron transfer process [7,8,35,38]. Bryce-Smith et al. and Covell and colleagues also observed the formation of the cyclobutane adduct upon irradiation in benzene, albeit only in trace amounts [35,39]. In this study, irradiation under standard conditions with UVB light produced significant amounts of phenanthrene due to competing excitation (**2e**: λ ≤ 300 nm [40]) and the subsequent oxidative electrocyclic photocyclization of **2e** [41]. In addition, *cis*-stilbene and other undefined compounds were noted in the ^1^H-NMR spectrum of the crude product. Consequently, even prolonged irradiation for 13 h resulted in a low conversion of **1** of just 40%, with an isolated yield of the spiro-oxetane **4e** of 27% (entry 5). When the **1**/**2e** pair was irradiated with 419 ± 25 nm light instead, the photoreaction proceeded cleanly, required a much shorter reaction time of 6 h, and achieved a high conversion of 93% (entry 6). From this reaction, the photoadduct **4e** was isolated in an improved yield of 78%. The structure of **4e** was confirmed by NMR spectroscopy. In particular, the ^1^H-NMR spectrum showed pairs of doublets for the oxetane protons at 4.64 and 6.27 ppm and the former quinonoid CH=CH protons at 6.48 ppm and 7.03 ppm, respectively. In addition, the ^13^C-NMR spectrum furnished three signals at 62.5, 79.0 and 81.4 ppm for the oxetane ring and one signal for the remaining C=O group at 184.2 ppm, respectively.

#### 2.1.3. Photocycloadditions with Diphenylacetylene

**Scheme 4:**
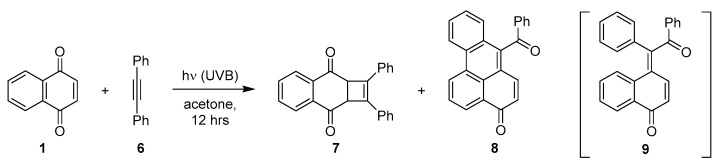
[2+2]-Photocycloaddition of 1,4-naphthoquinone with diphenylacetylene.

With the aim of expanding the reaction scope, the photoaddition of 1,4-naphthoquinone (**1**) with diphenylacetylene (**6**) was, furthermore, investigated (Scheme 4). Irradiation in acetone using Pyrex-filtered UVB light for 12 h resulted in the complete consumption of **1** and produced a mixture of the cyclobutene **7** in 13% and the benzoanthracenone **8** in 75%, respectively. Farid and colleagues noted that photocycloadditions involving alkynes utilize different excited states of the quinone [42]. Bosch et al. suggested that quinone methide (**9**) formation is initiated by either photoinduced electron transfer or direct excitation of the quinone [43]. Compound **8** was formed from the initial spiro-oxetene via ring-opening to **9** and subsequent photoinduced dehydrocyclization.

The results obtained are in general agreement with those reported by Chen et al., who isolated **8** and **7** in yields of 24% and 37% after 16 h of irradiation in benzene with light >400 nm [13]. Pappas and Portnoy also investigated this transformation and obtained a 1:4 mixture of the cyclobutene adduct **7** and the open methide product **9** after 9 h of irradiation with UVA light in acetonitrile [44]. The latter authors concluded that the competing modes of addition were impacted by the solvent, temperature and concentration.

**Figure 5:**
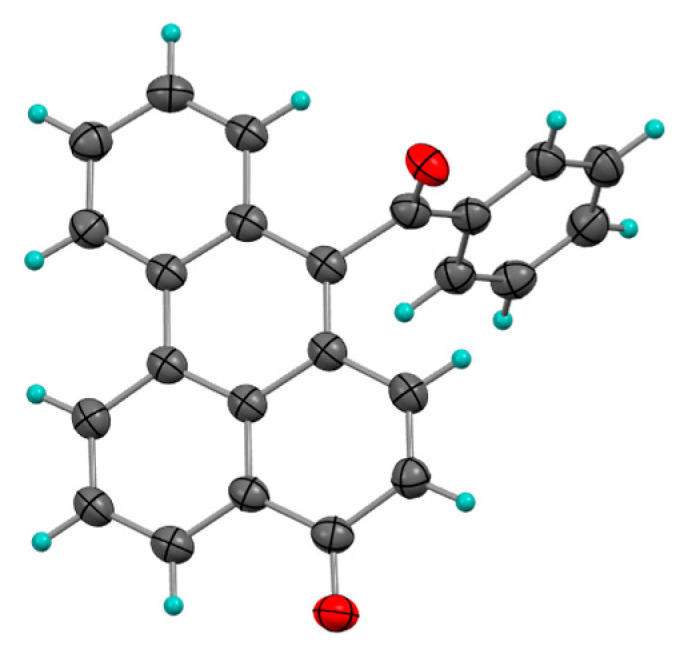
Crystal structure of **8** (from *n*-hexane/ethyl acetate).

The identity of the structures of **7** and **8** was confirmed by NMR analyses in comparison with the literature data for both compounds [42,44]. The ^1^H-NMR spectrum of **7** produced a singlet at 4.47 ppm, and the corresponding ^13^C-NMR spectrum gave a doublet at 50.9 ppm for the equivalent CH groups. Likewise, the ^1^H-NMR spectrum of **8** showed a pair of doublets for the olefinic protons at 6.69 and 7.61 ppm. Its structure was furthermore confirmed by X-ray crystallography (Figure 5). The four rings of the benzoanthracenone moiety formed a single plane, while the benzoyl group was twisted out of this plane by *ca* 86°.

### 2.2. Photocycloadditions Under Continuous-Flow Conditions

The batch conditions of the photocycloadditions were, consequently, applied to a continuous-flow mode in an in-house reactor system.

#### 2.2.1. In-House Capillary Reactor

**Figure 6:**
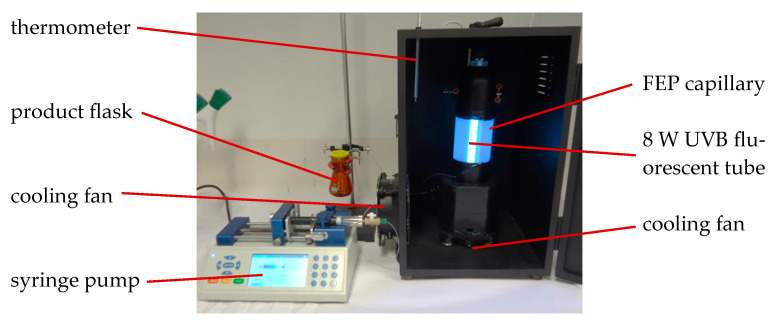
In-house continuous-flow capillary reactor.

The reactor (Figure 6) used a fluorinated ethylene propylene (FEP) capillary with an exposed length of 10 m, an inner diameter of 0.8 mm and an internal volume of 5 mL [45]. The capillary was wrapped around a Pyrex cylinder with an outer diameter of 6.5 cm (48 windings covering 7.8 cm). At its center, the reactor contained a single 8 W UVB fluorescence tube (inside-out irradiation). A small fan was mounted into the base of the reactor column to cool the lamp. The whole setup was kept in a light-tight cabinet. An additional cooling fan was mounted on the left side, and a thermometer was placed through the top of the cabinet for temperature control and monitoring during operation. An external syringe pump was used to transfer the reaction mixture through the capillary tubing. The product solution was collected outside the reactor box in an amber flask.

#### 2.2.2. Residence Time and Flow Rate Study

**Figure 7:**
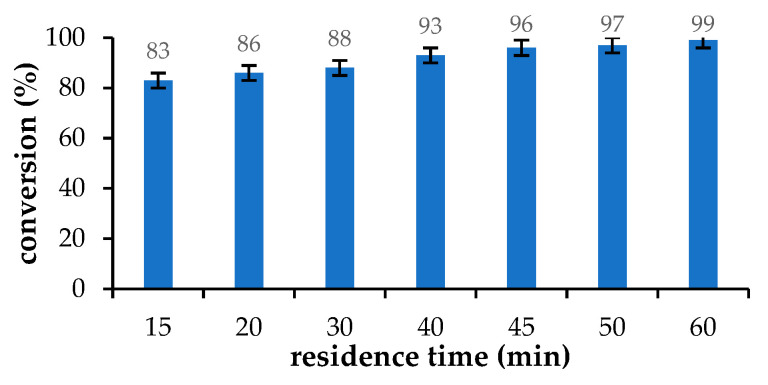
Conversion rates vs. residence time.

To find the optimal residence time for a high conversion rate, a series of flow reactions for the **1**/**2a** model pair were carried out again. Previously degassed acetone solutions of the starting materials were pumped through the flow reactor at a constant flow rate, and the effluent was continuously collected in an external flask. The use of acetone kept all the materials in solution, hence preventing the risk of clogging caused by solid materials [46]. The conversion rates were determined by ^1^H-NMR analysis of the crude product, and the results are depicted in Figure 7.

As expected, increasing the residence time resulted in a higher conversion. Initial irradiation with a residence time of 15 min gave a conversion of 83%, which gradually increased until a near completion rate of 99% with a residence time of 60 min was reached. By transferring the reaction protocol from batch to flow conditions, a significant reduction in reaction time was thus achieved.

#### 2.2.3. Photocycloadditions with Other Alkenes

The photocycloaddition protocol was subsequently extended to all alkenes (**2a**–**e**) (Scheme 3). To prevent photodegradation by overexposure, a generic residence time of 60 min was maintained. Under these conditions, acceptable to high conversions of 50–99% and yields of 47–84% were achieved (Table 4). Despite the competing photochemistry of *trans*-stilbene (**2e**) in UVB light [40,41], half of the naphthoquinone **1** was successfully consumed and the spiro-oxetane product **4e** was isolated in a compared to the batch process improved yield of 47% (entry 5).

#### 2.2.4. Photocycloadditions with Diphenylacetylene

The photoaddition of **1** with diphenylacetylene (**6**) was further investigated under flow conditions (Scheme 4). Using the standard residence time of 60 min, complete conversion was observed, and cyclobutene **7** and benzoanthracenone **8** were obtained with yields of 20% and 75%, respectively.

Despite its simple design, the continuous-flow photoreactor showed better performances in terms of reaction time and utilization of available light. Under flow conditions, exposure times were reduced from 10–13 h to 60 min. This was achieved by improving light penetration within the microcapillary in combination with the superior inside-out reactor design. In addition, the removal of the potentially photoactive products **3** and **4** from the irradiated zone further reduced or avoided any photodecompositions. These beneficial features consequently improved conversions, yields, product qualities and selectivity. Flow operation was also found to be more economical in terms of the light source applied. The flow reactor utilized a single 8 W fluorescent tube, while the much larger Rayonet chamber reactor employed 16 × 8 W tubes instead.

## 3. Materials and Methods

### 3.1. General Information

All solvents and reagents were commercially available (Merck Life Science Pty Ltd., Bayswater, VIC, Australia or Thermo Fisher Scientific Australia Pty Ltd, Scoresby VIC, Australia) and were used without purification. 1,4-Naphthoquinone was purified by sublimation prior to use.

Batch irradiation experiments were carried out in a Rayonet RPR-200 photochemical chamber reactor (Southern New England Ultraviolet Company, Branford, CT, USA) equipped with 16 × 8 W UVA, UVB or visible light (cool white or 419 ± 25 nm) fluorescent or UVC germicidal tubes. Pyrex or quartz Schlenk flasks with capacities of 60 mL were used as reaction vessels. A cold finger was inserted into the flask to maintain a reaction temperature below 25 °C. Photoreactions were monitored by thin-layer chromatography (TLC) or ^1^H-NMR spectroscopy. Continuous-flow irradiations were conducted in an in-house reactor system equipped with a single UVB fluorescent tube (Figure 6).

Structure determinations were undertaken on the MX1 beamline at the Australian Synchrotron, part of ANSTO [47]. Crystallographic data (excluding structure factors) for the structures reported in this paper have been deposited at the Cambridge Crystallographic Data Centre (CCDC) as supplementary numbers CCDC2345857 (*anti-***3a**), CCDC2345858 (**3b**), CCDC2345859 (*anti-***3c**), CCDC2345856 (*anti-***3d**) and CCDC2345864 (**8**). These data can be obtained free of charge from the CCDC.

### 3.2. Photocycloadditions

- General procedure for UVB photocycloadditions under batch conditions

A solution of 1,4-naphthoquinone (1 mmol) and alkene or alkyne (5 mmol) in acetone (50 mL) was irradiated in a Schlenk flask for 10–13 h while a slow, constant stream of nitrogen was passed through it. The crude reaction mixture was evaporated to dryness, and the residue was subjected to automated column chromatography using mixtures of cyclohexane and ethyl acetate or *n*-hexane and ethyl acetate as the mobile phase.

- General procedure for UVB photocycloadditions under continuous-flow conditions

The capillary of the continuous-flow reactor was filled with acetone, and the cooling fans and fluorescent tube were started. A solution of 1,4-naphthoquinone (0.5 mmol) and alkene or alkyne (2.5 mmol) in acetone (25 mL) was degassed with nitrogen for 5 min, loaded into a syringe and pumped at a flow rate of 0.083 mL/min through the in-house flow photoreactor equipped with a single 8 W UVB fluorescent tube (Ushio G8T5E). At the end of the delivery, the capillary was flushed with approx. 15 mL of acetone. The reaction mixture and acetone washings were collected in an amber round-bottom flask. The products were isolated as described above.

All photocyclization products **3a**–**d**, **4a** and **e**, **7** and **8** are known, and their spectroscopic details matched previously described data [25,26,35,44,48]. Characteristic spectroscopic details and NMR spectra of all the products are compiled in the Supplementary Materials.

## 4. Conclusions

In conclusion, a series of photocycloadditions of 1,4-naphthoquinone with alkenes and a model alkyne were successfully conducted. In most cases, simple irradiation in acetone with UVB light enabled the selective generation of cyclobutane photoadducts in acceptable to good yields without any noticeable photodegradation. With *trans*-stilbene, the isomeric spiro-oxetane was obtained instead, while irradiation in the presence of diphenylacetylene furnished a mixture of a cyclobutene and benzoanthracenone. When transferred to a continuous-flow reactor system, a significant reduction in reaction time from several hours to one hour, improved conversions and higher isolated yields were achieved. The simple reaction protocol may be subsequently scaled-up or conducted in a more advanced flow reactor [49,50].

## Figures and Tables

**Table 1:** Experimental results of the solvent optimization study (300 ± 25 nm, Pyrex, 10 h).

Entry	Solvent	Conversion (%) ^1^	Composition ^1^		Yield of 3a (%) ^2^
			3a (%)	4a (%)	
1	acetonitrile	88	93	7	51
2	trifluorotoluene	87	94	6	58
3	chloroform	100	78	22	55
4	acetone	96	100	0	69
5	methanol	100	photoreduction to **5**		n.d. ^3^

^1^ Determined by ^1^H-NMR analysis (±3%). ^2^ After automated flash chromatography. ^3^ Not determined.

**Table 2:** Experimental results of the wavelength optimization study (10 h).

Entry	Irradiation Conditions	Solvent	Conversion (%) ^1^	Composition ^1^	
				3a (%)	4a (%)
1	400–700 nm, ^2^ Pyrex	acetone	73	63	37
2	419 ± 25 nm, Pyrex	acetone	89	82	18
3	419 ± 25 nm, Pyrex	trifluorotoluene	74	81	19
4	419 ± 25 nm, Pyrex	acetonitrile	69	90	10
5	350 ± 25 nm, Pyrex	acetone	93	95	5
6	300 ± 25 nm, Pyrex	acetone	96	100	0
7	300 ± 25 nm, Quartz	acetone	96	100	0
8	254 nm, Quartz	acetone	91	100	0
9	254 nm, Quartz	acetonitrile	65	100	0

^1^ Determined by ^1^H-NMR analysis (±3%). ^2^ Cool white fluorescent tubes.

**Table 3:** Experimental results of photocyclizations with various alkenes under batch conditions with Pyrex-filtered UVB light.

Entry	Alkene (2)			Time (h)	Conversion (%) ^1^	Yield (%) ^2^
	R^1^	R^2^	R^3^			
1	Ph	H	H	10	96	69 (**3a**)
2	Ph	H	Ph	12	78	42 (**3b**)
3	-(CH_2_)_3_-		H	13	67	50 (**3c**)
4	-(CH_2_)_4_-		H	12	84	25 (**3d**)
5	H	Ph	Ph	13	40	27 (**4e**)
6 ^3^	H	Ph	Ph	6	93	78 (**4e**)

^1^ Determined by ^1^H-NMR analysis (±3%). ^2^ After automated flash chromatography. ^3^ Using 419 ± 25 nm light.

**Table 4:** Experimental results of photocyclizations with various alkenes under continuous-flow conditions (60 min).

Entry	Alkene (2)			Conversion (%) ^1^	Yield (%) ^2^
	R^1^	R^2^	R^3^		
1	Ph	H	H	99	84 (**3a**)
2	Ph	H	Ph	86	60 (**3b**)
3	-(CH_2_)_3_-		H	88	73 (**3c**)
4	-(CH_2_)_4_-		H	78	55 (**4d**)
5	H	Ph	Ph	50	47 (**4e**)

^1^ Determined by ^1^H-NMR analysis (±3%). ^2^ After automated flash chromatography.

## Data Availability

All data is available on request from the corresponding author.

## Supplementary Materials

The following supporting information can be downloaded at: https://www.mdpi.com/article/10.3390/molecules29245920/s1, Experimental details, procedures, spectroscopic data and NMR spectra; Figure S1: Photocycloaddition in the Rayonet reactor during irradiation; Figure S2: In-house continuous-flow capillary reactor during operation.
