# Supplementary material for: [2+2]-Photocycloadditions of 1,4-Naphthoquinone Under Batch and Continuous-Flow Conditions
Source: Molecules. 2024 Dec 15;29(24):5920. doi: 10.3390/molecules29245920 (PMC11676797; doi:10.3390/molecules29245920)
Supplement: Supplementary file 1 [file molecules-29-05920-s001.zip › molecules-3361114-supplementary.pdf]

Supplementary Materials

[2+2]-Photocycloadditions of 1,4-Naphthoquinone Under Batch and Continuous-Flow Conditions

Madyan A. Yaseen [1,2], Zhifang Guo [1], Peter C. Junk [1] and Michael Oelgemöller [1,3,*]

[1] College of Science and Engineering, James Cook University, Townsville, QLD 4811, Australia
[2] College of Education, University of Samarra, Samarra 34010, Salah Al-Deen, Iraq; madyan.yaseen@uosamarra.edu.iq
[3] Faculty of Chemistry and Biology, Hochschule Fresenius gGmbH—University of Applied Science,
65510 Idstein, Germany
* Correspondence: michael.oelgemoeller@jcu.edu.au

Experimental

General methods

Solvents and reagents

All solvents and reagents were commercially available (Merck Life Science Pty Ltd, Bayswater, VIC, Australia or Thermo Fisher Scientific Australia Pty Ltd, Scoresby VIC, Australia) and were used without purification. 1,4-Naphthoquinone was purified by sublimation prior to use.

Photochemical equipment and procedures

Batch irradiation experiments were carried out in a Rayonet RPR-200 photochemical chamber reactor (Southern New England Ultraviolet Company, Branford, CT, USA) equipped with 16 × 8 W UVA (350 ± 25 nm), UVB (300 ± 25 nm), visible light (cool white or 419 ± 25 nm) or UVC (254 nm) fluorescent or germicidal tubes. Pyrex ($\lambda \geq 300$ nm) or quartz ($\lambda \geq 200$ nm) Schlenk flasks with capacities of approx. 60 mL were used as reaction vessels. A cold finger was inserted into the flask to maintain the reaction temperature below 25°C. The reaction mixtures were degassed with N_2 through a sidearm for approx. 5 min prior to and constantly during irradiations. Photoreactions were monitored by thin-layer chromatography (TLC) or ^{1}H-NMR spectroscopy. The crude reaction mixture was evaporated to dryness and the residues were subjected to automated column chromatography using gradient mixtures of cyclohexane and ethyl acetate or *n*-hexane and ethyl acetate as mobile phase.

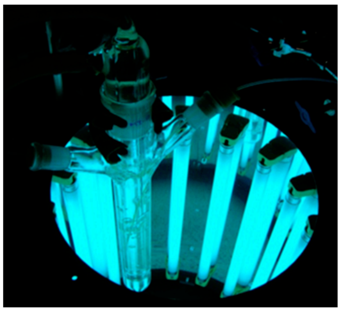

Figure S1. Photocycloaddition in the Rayonet reactor during irradiation.

Continuous-flow irradiations were conducted in an in-house reactor system. The reactor used a fluorinated ethylene propylene (FEP, Bohlender GmbH) capillary with an exposed length of 10 m, an inner diameter of 0.8 mm and an internal volume of 5 mL. The capillary was wrapped around a Pyrex cylinder with an outer diameter of 6.5 cm (48 windings covering 7.8 cm). At its center, the reactor contained a single 8 W UVB fluorescence tube (inside-out irradiation). A small fan was mounted into the base of the reactor column to cool the lamp. The whole setup was kept in a light-tight cabinet. An additional cooling fan was mounted on the left side and a thermometer through the top of the cabinet for temperature control and monitoring during operation. An external syringe pump (Chemyx model Fusion 200) was used to transfer the reaction mixture through the capillary tubing. The capillary of the continuous-flow reactor was filled with acetone and the cooling fans and fluorescent tube were started. A solution of 1,4-naphthoquinone (0.5 mmol) and alkene or alkyne (2.5 mmol) in acetone (25 mL) was degassed with nitrogen for 5 minutes, loaded into a syringe and pumped at a flow rate of 0.083 mL/min through the in-house flow photoreactor equipped with a single 8 W UVB fluorescent tube. At the end of the delivery the capillary was flushed with approx. 15 mL of acetone. The reaction mixture and acetone washings were collected in an amber round-bottom flask. The products were isolated as described above.

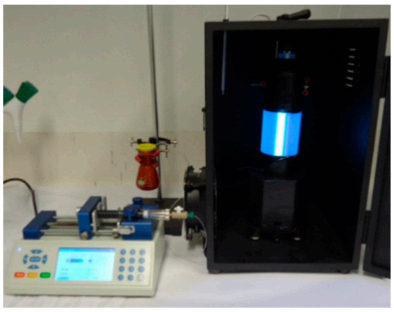

Figure S2. In-house continuous-flow capillary reactor during operation.

Chromatographic methods

Thin layer chromatography (TLC) was completed in glass jars on silica gel plates (Macherey-Nagel polygram sil G UV254). Products were visualized with UV irradiation at 365 or 254 nm, respectively. Column chromatography was carried out in Pyrex glass columns using Scharlau silica gel 60 (particle size 0.06-0.2 nm) 70-230 mesh ASTM as a stationary phase. A CombiFlash® Rf+ Lumen™ flash chromatography system from Teledyne Isco was used to purify product mixtures. The system recorded UV spectra in real-time during peak elution. Normal phase cartridges were used. Mixtures of ethyl acetate and cyclohexane or ethyl acetate and *n*-hexane were used as the mobile phase.

Analytical methods

Melting points were measured in open capillaries using a Tathastu or Gallenkamp melting point apparatus and are uncorrected.

NMR spectra were recorded on a Bruker 400 Ascend™ (^{1}H: 400 MHz and ^{13}C: 100 MHz) equipped with an auto-sampler. NMR spectra were processed using the MestReNova software and the residual solvent peak as internal standard. Samples were prepared in $CDCl_3$ (δ = 7.26/77.3 ppm ppm) and acetone-d_6 (δ = 2.09/30.6 ppm). Chemical shifts (δ) are reported in ppm, coupling constants in Hertz (Hz). Multiplicities were reported as s (singlet), d (doublet), t (triplet), q (quartet), quin (quintet), sxt (sextet), sept (septet), m (multiplet) and br (broad).

Infrared spectra were recorded on a Perkin Elmer Spectrum One FT-IR Spectrometer as thin films. Spectra were recorded in the range 600-4000 cm^{-1}.

Mass spectra were recorded using direct injection on a Shimadzu LCMS-2020 equipped with a DUIS ion source. Ions were subsequently detected in positive mode and/or negative mode within a mass range of m/z 100-500. The mobile phase were aqueous solutions of HPLC grade methanol or acetonitrile with 0.1% formic acid added. All experimental event sequences were controlled, and processing performed using LabSolutions for LCMS-2020 software.

For X-ray crystallographic analysis, crystals were immersed in crystallography oil and were measured on the MX1 beamline at the Australian Synchrotron. Data integration was completed using Blue-ice[1] and XDS[2] software programs. Structural solutions were obtained by SHELXT intrinsic phasing method and refined using full-matrix least-squares methods against F^2 using SHELX2015,[3] in conjunction with Olex2[4] graphical user interface. All hydrogen atoms were placed in calculated

positions using the riding model. Crystal data and refinement details for compounds *anti*-**3a**, **3b**, *anti*-**3c**, *anti*-**3d** and **8** are given in **Table S1**. Their corresponding CCDC files contain the supplementary crystallographic data. These data can be obtained free of charge from The Cambridge Crystallographic Data Centre via www.ccdc.cam.ac.uk/data_request/cif.

Table S1. Crystal data and structural refinement for compounds *anti*-**3a**, **3b**, *anti*-**3c**, *anti*-**3d** and **8**.

	anti-**3a**	**3b**	*anti*-**3c**	*anti*-**3d**	**8**
**CCDC No.**	2345857	2345858	2345859	2345856	2345864
**Formula**	$C_{18}H_{14}O_2$	$C_{24}H_{18}O_2$	$C_{15}H_{14}O_2$	$C_{16}H_{16}O_2$	$C_{24}H_{14}O_2$
M_r	262.29	338.38	226.26	240.29	334.35
**Crystal System**	Monoclinic	Monoclinic	Monoclinic	Monoclinic	Orthorhombic
**Space group**	*C*2/c	*P*2$_1$/n	*P*2$_1$/c	*P*2$_1$/c	*P*bca
a **(Å)**	32.872(7)	9.1680(18)	9.1860(18)	9.7000(19)	9.980(2)
b **(Å)**	9.2550(19)	16.291(3)	11.527(2)	11.577(2)	14.490(3)
c **(Å)**	23.836(5)	11.981(2)	10.444(2)	10.597(2)	22.650(5)
α **(°)**	90	90	90	90	90
β **(°)**	133.42(3)	107.89(3)	90.31(3)	91.52(3)	90
γ **(°)**	90	90	90	90	90
V **(Å^3)**	5267(3)	1702.9(6)	1105.9(4)	1189.6(4)	3275.4(11)
Z	16	4	4	4	8
ρ_{calc}, **g cm^{-3}**	1.323	1.320	1.359	1.342	1.356
μ, **mm^{-1}**	0.085	0.083	0.089	0.087	0.085
N_τ	29573	19262	12793	13790	72300
N (R_{int})	4580 (0.0536)	2734 (0.0184)	1837 (0.0238)	2074 (0.0317)	2877 (0.0365)
R_1 $(I > 2\sigma(I))$	0.1035	0.0376	0.0382	0.0395	0.0387
wR_2 **(all data)**	0.3159	0.0989	0.1022	0.1026	0.1028
GOF	1.119	1.061	1.057	1.058	1.062

Spectroscopic data

All compounds synthesized are known and their spectroscopic details matched previously described data.[5-10]

1-Phenyl-1,2,2a,8a-tetrahydrocyclobuta[b]naphthal-ene-3,8-dione (3a) [5-7]

anti-**3a**: Colourless crystals. M.p.: 115-116°C. ^{1}H-NMR (400 MHz, $CDCl_3$): δ = 8.10 (dd, J = 9.3 Hz, 2H, CH_{arom}), 7.73 (dd, J = 5.3 Hz, 2H, CH_{arom}), 7.26 (dd, J = 5.3 Hz, 4H, CH_{arom}), 7.17 (d, J = 6.9 Hz, 1H, CH_{arom}), 3.84–3.67 (m, 2H, CH), 3.56–3.40 (m, 1H, CH), 2.77 (dd, J = 10.7 Hz, 1H, CH), 2.68 (ddd, J = 12.0, 7.7, 3.3 Hz, 1H, CH). ^{13}C-NMR (100 MHz, $CDCl_3$): δ = 198.6 (s, 1C, C=O), 195.4 (s, 1C, C=O), 142.2 (s, 1C, Cq_{arom}), 135.6 (s, 1C, Cq_{arom}), 135.2 (s, 1C, Cq_{arom}), 134.6 (d, 1C, CH_{arom}), 134.4 (d, 1C, CH_{arom}), 128.7 (d, 2C, 2×CH_{arom}), 127.6 (d, 1C, CH_{arom}), 127.4 (d, 1C, CH_{arom}), 126.9 (d, 1C, CH_{arom}), 126.3 (d, 2C, 2×CH_{arom}), 51.3 (d, 1C, CH_{arom}), 44.7 (d, 1C, CH), 41.1 (t, 1C, CH_2), 32.2 (s, 1C, CH). MS (DUIS): m/z = 263 [M^++H]; expected: 262 [M^+]. IR (neat): ῡ = 1671, 1587, 1449, 1283, 1262, 1119, 968, 761.

syn-**3a**: Colourless solid. ^{1}H-NMR (400 MHz, $CDCl_3$): δ = 8.12 (dd, J = 7.7, 1.0 Hz, 1H, CH_{arom}), 7.86 (dd, J = 7.7, 1.1 Hz, 1H, CH_{arom}), 7.75 (ddd, J = 7.6, 1.4 Hz, 1H, CH_{arom}), 7.68 (ddd, J = 7.5, 1.4 Hz, 1H, CH_{arom}), 7.23–7.13 (m, 2H, CH_{arom}), 7.03 (d, J = 6.9 Hz, 2H, CH_{arom}), 4.24 (dd, J = 19.8, 9.2 Hz, 1H, CH), 4.02 (ddd, J = 9.0, 8.0, 2.9 Hz, 1H, CH), 3.75 (ddd, J = 9.8, 8.0 Hz, 1H, CH), 2.99 (ddd, J = 12.0, 10.4, 2.0 Hz, 1H, CH), 2.80 (ddd, J = 13.0, 9.2, 0.8 Hz, 1H, CH). ^{13}C-NMR (100 MHz, $CDCl_3$): δ = 197.3 (s, 1C, C=O), 195.2 (s, 1C, C=O), 138.4 (s, 1C, Cq_{arom}), 136.8 (s, 1C, Cq_{arom}), 134.9 (s, 1C, Cq_{arom}), 134.4 (d, 1C, CH_{arom}), 134.3 (d, 1C, CH_{arom}), 128.4 (s and d, 2C, Cq_{arom} and CH_{arom}), 128.0 (d, 2C, 2×CH_{arom}), 127.5 (d, 1C, CH_{arom}), 127.2 (d, 2C, 2×CH_{arom}), 50.1 (d, 1C, CH), 42.4 (d, 1C, CH), 40.7 (t, 1C, CH_2), 31.2 (d, 1C, CH).

1,1-Diphenyl-1,2,2a,8a-tetrahydrocyclobuta[b]naphthalene-3,8-dione (3b) [6]

Colourless crystals. M.p.: 136-137°C. ^{1}H-NMR (400 MHz, $CDCl_3$): δ = 7.96 (dd, J = 7.7, 1.7 Hz, 1H, CH_{arom}), 7.80 (dd, J = 7.6, 1.2 Hz, 1H, CH_{arom}), 7.56 (ddd, J = 7.4, 1.5 Hz, 2H, 2×CH_{arom}), 7.48 (dd, J = 8.3, 1.2 Hz, 2H, 2×CH_{arom}), 7.37 (dd, J = 7.8 Hz, 2H, 2×CH_{arom}), 7.22 (dd, J = 7.4 Hz, 1H, CH_{arom}), 7.09 (dd, J = 8.5, 1.2 Hz, 2H, 2×CH_{arom}), 6.95 (dd, J = 7.7 Hz, 2H, 2×CH_{arom}), 6.84 (dd, J = 7.3 Hz, 1H, CH_{arom}), 4.62 (d, J = 8.8 Hz, 1H, CH), 3.68 (dd, J = 12.5, 2.9 Hz, 1H, CH), 3.56 (ddd, J = 10.7, 8.8, 2.9 Hz, 1H, CH), 3.13 (dd, J = 12.5, 10.6 Hz, 1H, CH). ^{13}C-NMR (100 MHz, acetone-d_6): δ = 198.7 (s, 1C, C=O), 195.8 (s, 1C, C=O), 151.2 (s, 1C, Cq_{arom}), 142.0 (s, 1C, Cq_{arom}), 137.3 (d, 1C, CH_{arom}), 136.8 (d, 1C, CH_{arom}), 134.7 (s, 1C, Cq_{arom}), 134.6 (s, 1C, Cq_{arom}), 129.3 (d, 4C, 4×CH_{arom}), 128.7 (d, 4C, 4×CH_{arom}), 127.2-127.0 (d, 4C,

4×CH_{arom}), 58.1 (d, 1C, CH), 55.1 (d, 1C, CH), 41.4 (d, 1C, CH), 36.9 (d, 1C, CH). MS (DUIS): m/z = 338 [M^+]; expected: 338 [M^+]. IR (neat): ῡ = 1675, 1586, 1494, 1287, 1270, 761, 752.

2,3,3a,3b,9a,9b-Hexahydro-1H-cyclopenta[3,4]cyclobuta[1,2-b]naphthalene-4,9-dione (*anti*-3c) [5,6]

Colourless needles. M.p.: 123-124°C. ^{1}H-NMR (400 MHz, $CDCl_3$): δ = 8.12 (d, J = 9.2 Hz, 2H, CH_{arom}), 7.77 (d, J = 9.2 Hz, 2H, CH_{arom}), 3.08 (d, J = 3.3 Hz, 2H, CH), 2.90 (d, J = 4.4 Hz, 2H, CH), 2.06–1.83 (t, 4H, CH), 1.62 (s, 2H, CH). ^{13}C-NMR (100 MHz, $CDCl_3$): δ = 197.6 (s, 2C, 2×C=O), 135.6 (s, 2C, 2×Cq_{arom}), 134.4 (d, 2C, 2×CH_{arom}), 127.5 (d, 2C, 2×CH_{arom}), 47.1 (d, 2C, 2×CH), 43.5 (d, 2C, 2×CH), 33.2 (t, 2C, 2×CH_2), 24.6 (t, 1C, CH_2). MS (DUIS): m/z = 227 [M^++H]; expected: 226 [M^+]. IR (neat): ῡ = 1673, 1583, 1320, 1274, 1262, 1228, 955, 788.

1,2,3,4,4a,4b,10a,10b-Octahydrobenzo[b]biphenylene-5,10-dione (*anti*-3d) [5,6]

Colourless crystals. ^{1}H-NMR (400 MHz, $CDCl_3$): δ = 8.13 (dd, J = 5.8, 3.3 Hz, 2H, 2×CH_{arom}), 7.76 (dd, J = 5.9, 3.3 Hz, 2H, 2×CH_{arom}), 3.35 (dd, J = 3.2, 1.4 Hz, 2H, 2×CH), 2.69 (board s, 2H, 2×CH), 1.84 (board s, 2H, CH_2), 1.61 (board s, 6H, 3×CH_2). ^{13}C-NMR (100 MHz, $CDCl_3$): δ = 197.8 (s, 2C, 2×C=O), 135.3 (s, 2C, 2×Cq_{arom}), 134.4 (d, 2C, 2×CH_{arom}), 127.4 (d, 2C, 2×CH_{arom}), 47.5 (d, 2C, 2×CH), 39.4 (d, 2C, 2×CH), 27.5 (t, 2C, 2×CH_2), 22.3 (t, 2C, 2×CH_2).

3',4′-Diphenyl-4H-spiro[naphthalene-1,2'-oxetan]-4-one (4e) [5]

Colourless needles. M.p.: 142-143°C. ^{1}H-NMR (400 MHz, $CDCl_3$): δ = 8.13 (ddd, J = 7.4, 5.1, 1.0 Hz, 2H, CH_{arom}), 7.73 (ddd, J = 8.0, 1.6 Hz, 1H, CH_{arom}), 7.62 (d, J = 7.6 Hz, 2H, 2×CH_{arom}), 7.55–7.47 (m, 3H, 3×CH_{arom}), 7.44–7.35 (m, 2H, 2×CH_{arom}), 7.33–7.25 (m, 3H, 3×CH_{arom}), 7.03 (d, J = 6.9 Hz, 2H, 2×CH_{arom}), 6.48 (d, J = 9.2 Hz, 1H, CH_{arom}), 6.27 (d, J = 10.4 Hz, 1H, CH), 4.64 (d, J = 9.3 Hz, 1H, CH). ^{13}C-NMR (100 MHz, $CDCl_3$): δ = 184.2 (s, 1C, C=O), 147.4 (d, 1C, CH_{arom}), 144.3 (s, 1C, Cq_{arom}), 141.2 (s, 1C, Cq_{arom}), 135.2 (s, 1C, Cq_{arom}), 133.7 (d, 1C, CH_{arom}), 130.2 (s, 1C, Cq_{arom}), 129.7 (d, 1C, CH_{arom}), 129.0 (d, 2C, 2×CH_{arom}), 129.0 (d, 1C, CH_{arom}), 128.5 (d, 1C, CH_{arom}), 127.9 (d, 1C, CH_{arom}), 127.7 (d, 1C, CH_{arom}), 127.1 (d, 2C, 2×CH_{arom}), 126.5 (d, 1C, CH_{arom}), 125.3 (d, 2C, 2×CH_{arom}), 81.4 (s, 1C, $Cq_{oxetane}$), 79.0 (d, 1C, $CH_{oxetane}$), 62.5 (d, 1C, $CH_{oxetane}$). MS (DUIS): m/z = 339 [M^++H]; expected: 338 [M^+]. IR (neat): ῡ = 1667, 1599, 1495, 1448, 1297, 1000, 966.

1,2-Diphenylcyclobuta[b]naphthalene-3,8(2aH, 8aH)-dione (7) [8,9]

Yellow solid. M.p.: 180-181°C. ^{1}H-NMR (400 MHz, $CDCl_3$): δ = 8.2 (dd, 2H, 2×CH_{arom}), 7.72 (dd, 2H, 2×CH_{arom}), 7.60 (dd, 4H, 4×CH_{arom}), 7.36–7.30 (m, 6H, 6×CH_{arom}), 4.47 (s, 2H, 2×CH). ^{13}C-NMR (100

MHz, $CDCl_3$): δ = 195.9 (s, 2C, 2×C=O), 141.1 (s, 2C, 2×Cq_{arom}), 134.6 (s, 2C, 2×Cq_{arom}), 134.2 (s, 2C, 2×Cq_{arom}), 133.4 (d, 2C, 2×CH_{arom}), 129.1 (d, 2C, 2×CH_{arom}), 128.7 (d, 4C, 4×CH_{arom}), 127.7 (d, 4C, 4×CH_{arom}), 127.1 (d, 2C, 2×CH_{arom}), 50.9 (s, 2C, 2×CH). MS (DUIS): m/z = 337 [M^++H]; expected: 336 [M^+]. IR (neat): ῡ = 1640, 1594, 1301, 1252, 1211, 1030, 700.

7-Benzoyl-4H-benzo[de]anthracen-4-one (8) [10]

Yellow crystals. M.p.: 209-210°C. ^{1}H-NMR (400 MHz, $CDCl_3$): δ = 9.05 (dd, J = 8.4, 1.0 Hz, 1H, H_{arom}), 8.80 (d, J = 8.4 Hz, 1H, H_{arom}), 8.71 (dd, J = 7.5, 1.2 Hz, 1H, H_{arom}), 8.02–7.95 (m, 1H, H_{arom}), 7.91 (d, J = 7.1 Hz, 2H, 2×H_{arom}), 7.79 (ddd, J = 8.3, 7.0, 1.3 Hz, 1H, H_{arom}), 7.71 (dd, J = 8.2, 0.7 Hz, 1H, H_{arom}), 7.67–7.54 (m, 3H, 3×H_{arom}), 7.48 (dd, J = 7.9 Hz, 2H, 2×H_{arom}), 6.69 (d, J = 10.0 Hz, 1H, H_{arom}). ^{13}C-NMR (100 MHz, $CDCl_3$): δ = 198.3 (s, 1C, C=O), 185.3 (s, 1C, C=O), 142.0 (s, 1C, Cq_{arom}), 141.5 (s, 1C, Cq_{arom}), 138.4 (s, 1C, CH_{arom}), 137.6 (s, 1C, Cq_{arom}), 134.9 (d, 1C, CH_{arom}), 131.5 (s, 1C, Cq_{arom}), 130.6 (s, 1C, Cq_{arom}), 130.4 (d, 1C, CH_{arom}), 130.2 (d, 1C, CH_{arom}), 130.0 (d, 1C, CH_{arom}), 129.8 (d, 1C, CH_{arom}), 129.8 (s, 1C, Cq_{arom}), 129.3 (d, 2C, 2×CH_{arom}), 129.1 (s, 1C, Cq_{arom}), 128.6 (d, 1C, CH_{arom}), 128.3 (d, 1C, CH_{arom}), 128.3 (d, 1C, CH_{arom}), 128.1 (d, 1C, CH_{arom}), 126.8 (s, 1C, Cq_{arom}), 123.6 (d, 1C, CH_{arom}), 123.4 (s, 1C, Cq_{arom}). MS (DUIS): m/z = 336 [M^++2H]; expected: 334 [M^+]. IR (neat): ῡ = 1660, 1631, 1579, 1251, 1221, 1174, 707.

^{1}H- and ^{13}C-NMR spectra

1-Phenyl-1,2,2a,8a-tetrahydrocyclobuta[b]naphthal-ene-3,8-dione (3a) [5-7]

Main *anti*-isomer (*anti*-3a): ^{1}H-NMR (400 MHz, CDCl$_3$):

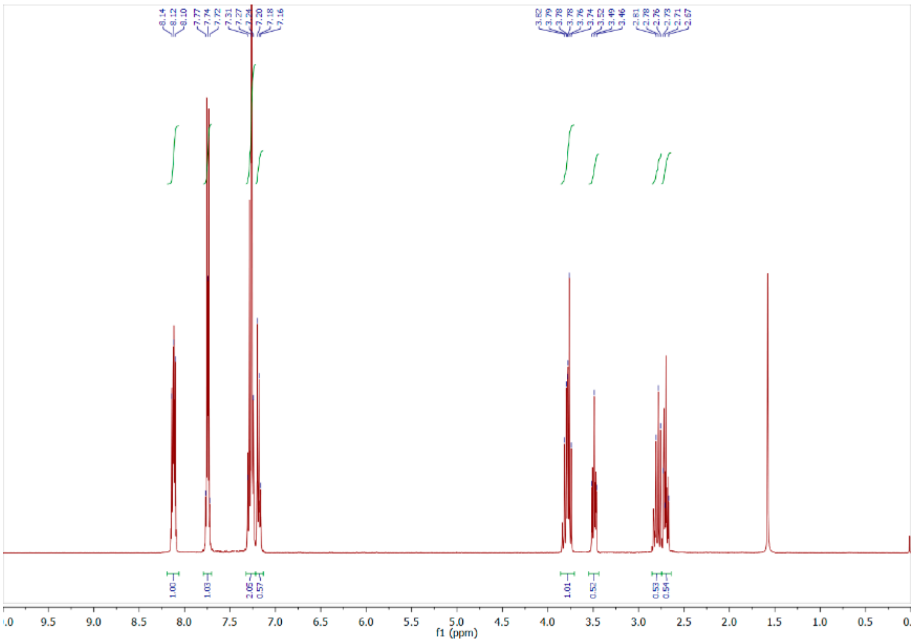

^{13}C-NMR (100 MHz, CDCl$_3$):

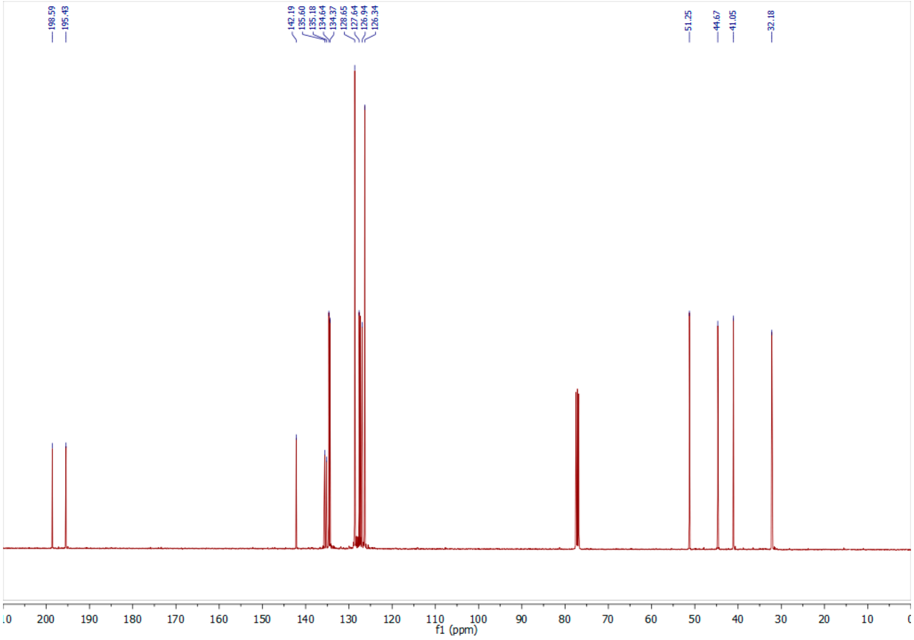

Minor *syn*-isomer: ^{1}H-NMR (400 MHz, $CDCl_3$):

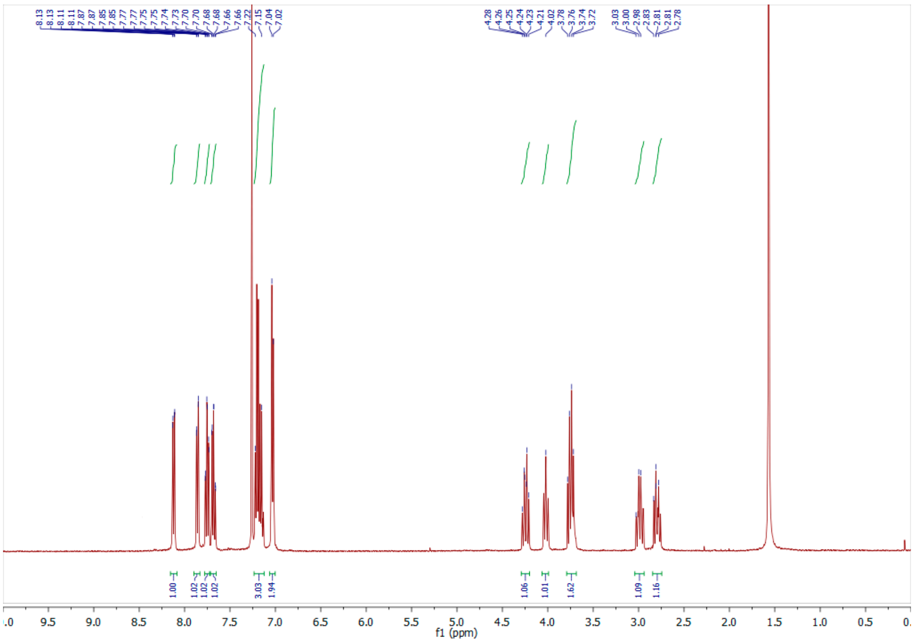

^{13}C-NMR (100 MHz, $CDCl_3$):

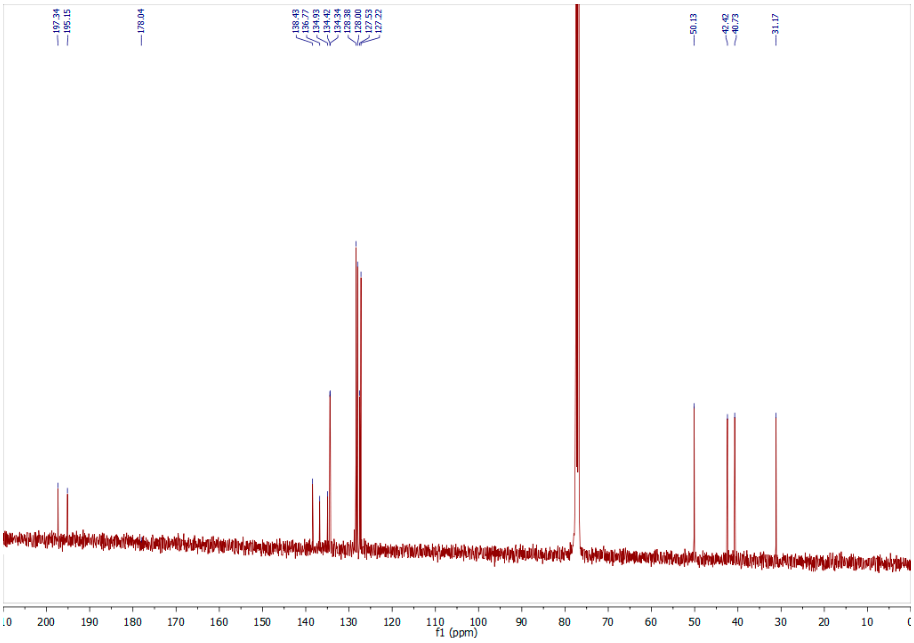

1,1-Diphenyl-1,2,2a,8a-tetrahydrocyclobuta[b]naphthalene-3,8-dione (3b) [6]

^{1}H-NMR (400 MHz, $CDCl_3$):

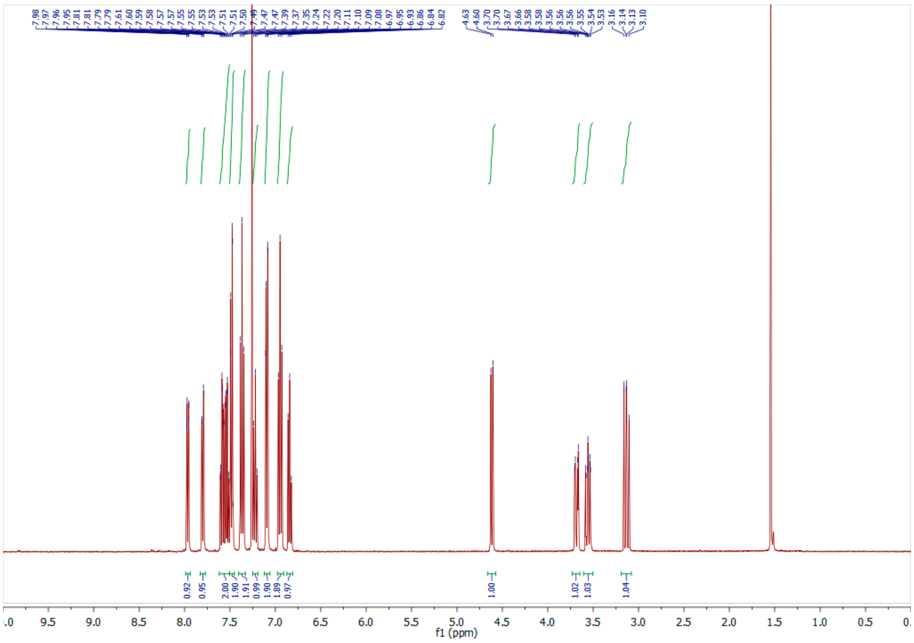

^{13}C-NMR (100 MHz, acetone-d_6):

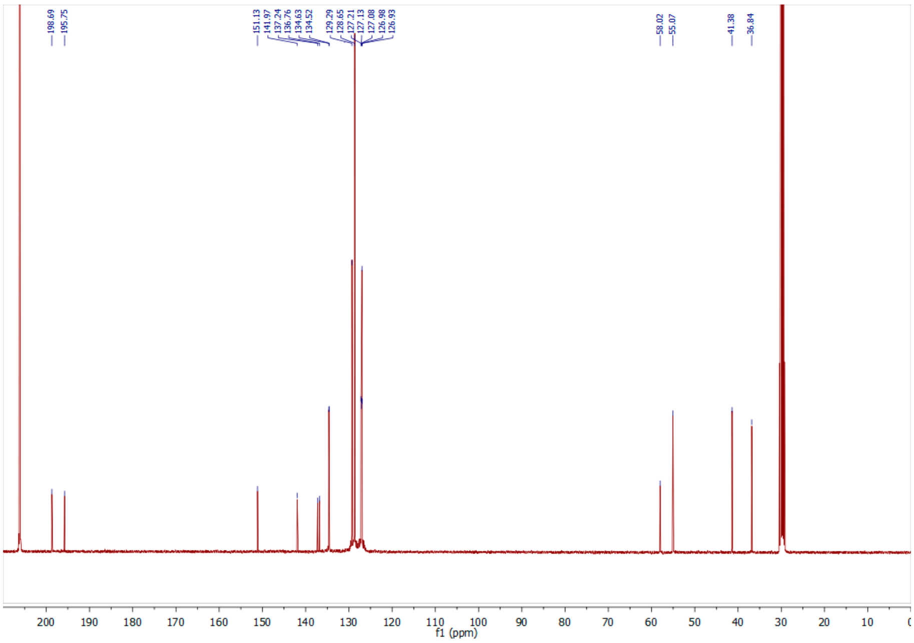

2,3,3a,3b,9a,9b-Hexahydro-1H-cyclopenta[3,4]cyclobuta[1,2-b]naphthalene-4,9-dione (*anti*-3c) [5,6]

^{1}H-NMR (400 MHz, $CDCl_3$):

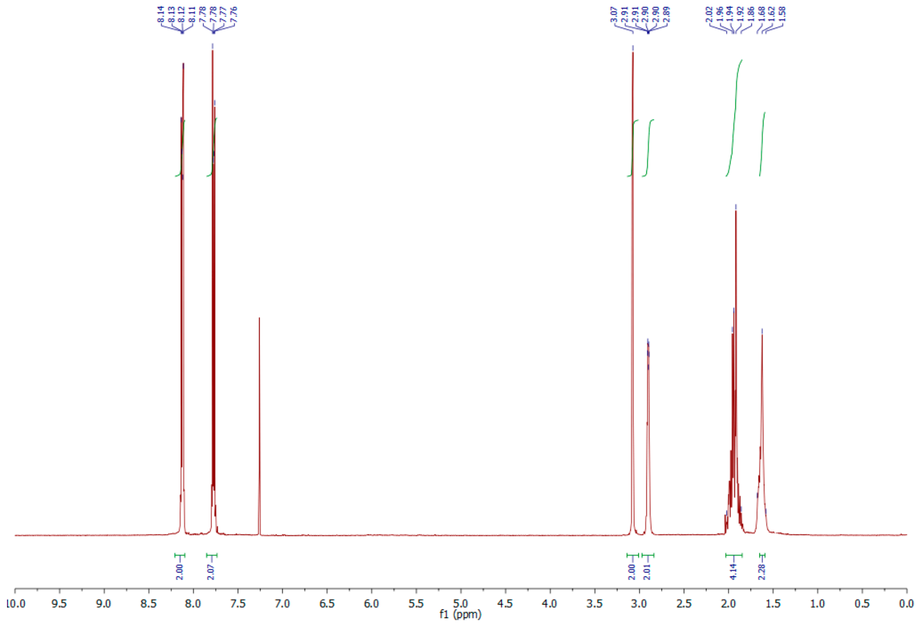

^{13}C-NMR (100 MHz, $CDCl_3$):

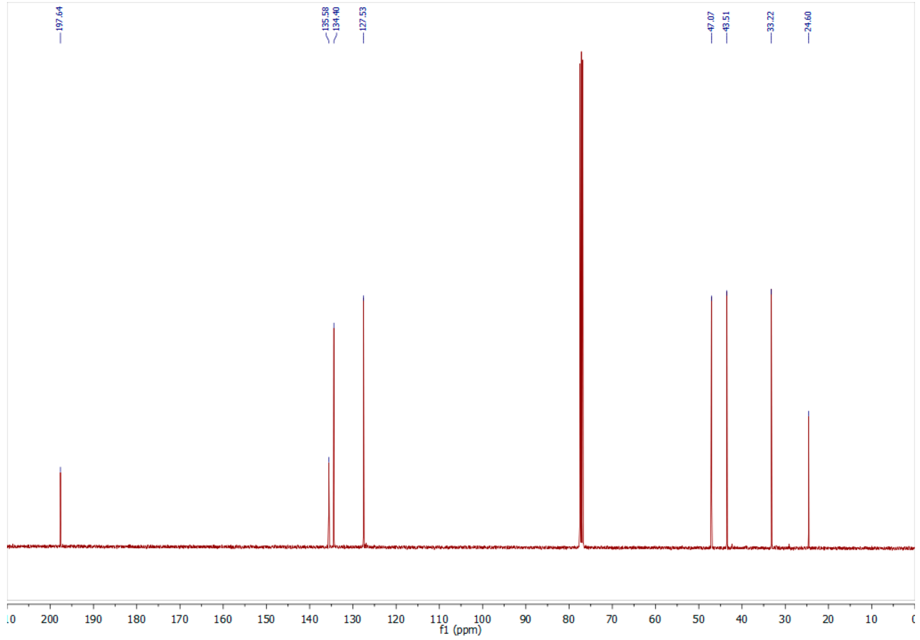

1,2,3,4,4a,4b,10a,10b-Octahydrobenzo[b]biphenylene-5,10-dione (*anti*-3d) [5,6]

^{1}H-NMR (400 MHz, $CDCl_3$):

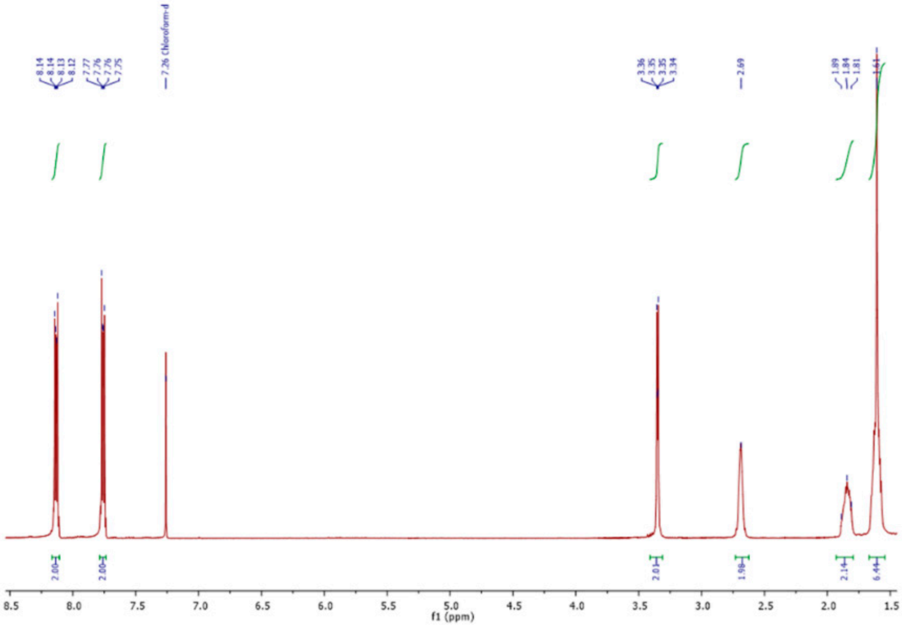

^{13}C-NMR (100 MHz, $CDCl_3$):

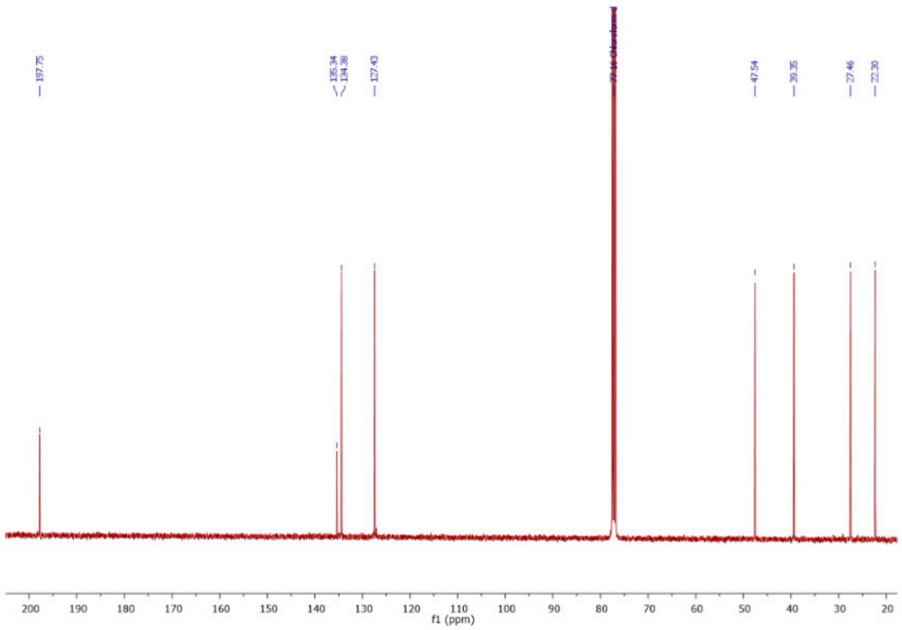

3',4'-Diphenyl-4H-spiro[naphthalene-1,2'-oxetan]-4-one (4e) [5]

^{1}H-NMR (400 MHz, CDCl$_3$):

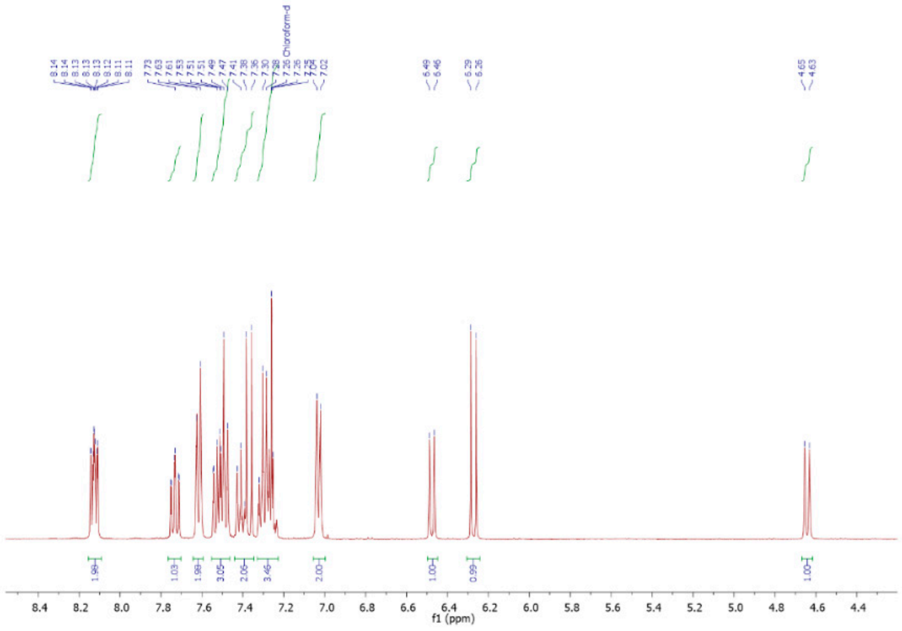

^{13}C-NMR (100 MHz, CDCl$_3$):

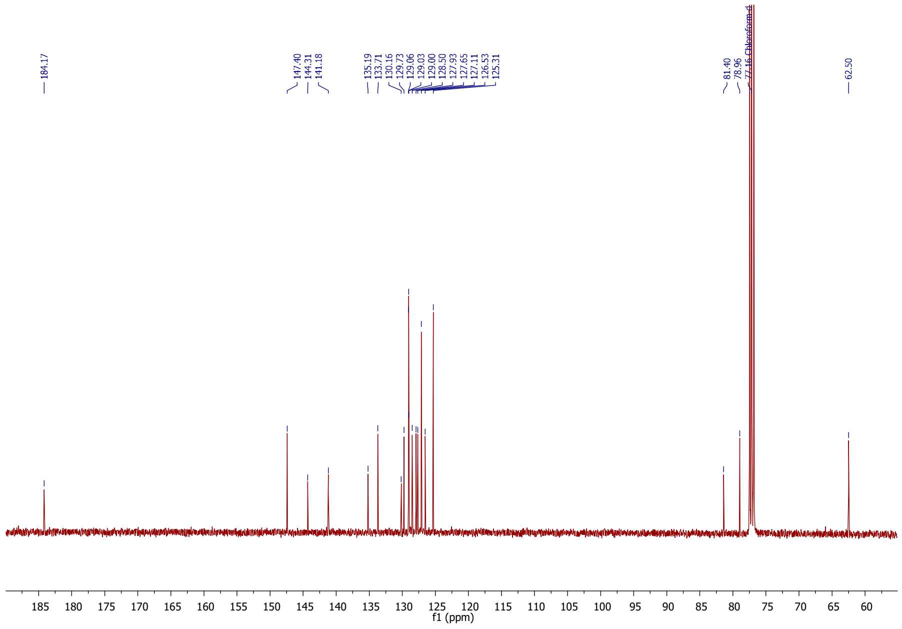

1,2-Diphenylcyclobuta[b]naphthalene-3,8(2aH, 8aH)-dione (7) [8,9]

^{1}H-NMR (400 MHz, CDCl$_3$):

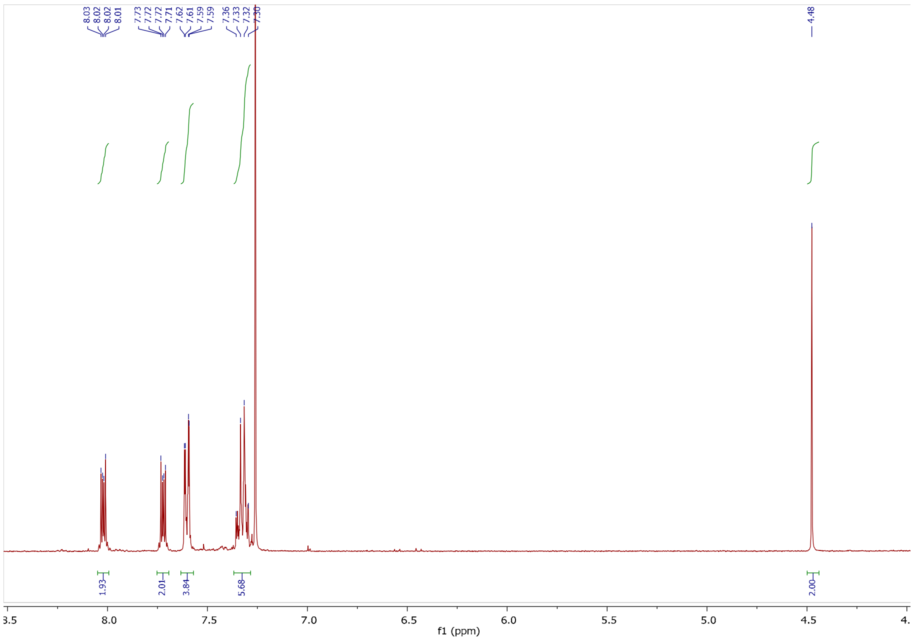

^{13}C-NMR (100 MHz, CDCl$_3$):

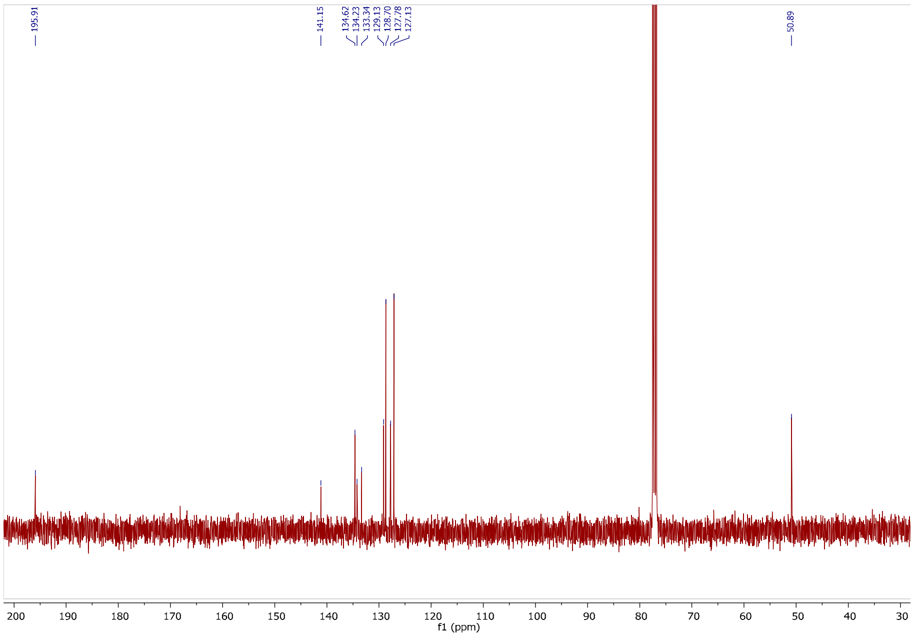

7-Benzoyl-4H-benzo[de]anthracen-4-one (8) [10]

^{1}H-NMR (400 MHz, CDCl$_3$):

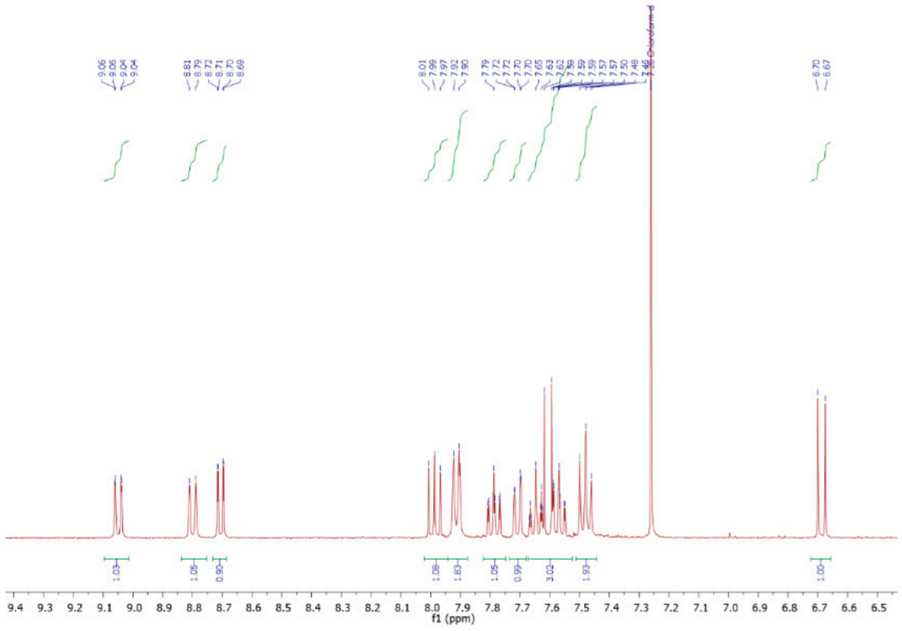

^{13}C-NMR (100 MHz, CDCl$_3$):

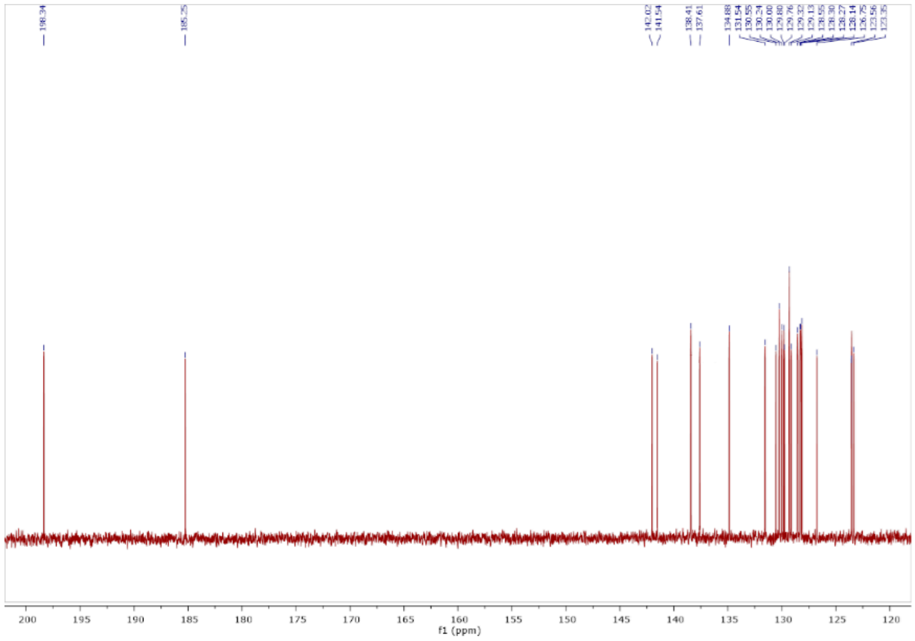

References

1. McPhillips, T.M.; McPhillips, S.E.; Chiu, H.J.; Cohen, A.E.; Deacon, A.M.; Ellis, P.J.; Garman, E.; Gonzalez, A.; Sauter, N.K.; Phizackerley, R.P.; Soltis, S.M.; Kuhn, J.P. Blu-Ice and the Distributed Control System: software for data acquisition and instrument control at macromolecular crystallography beamlines. *J. Synchrotron Radiat*. **2002**, *9*, 401–406.
2. Kabsch, W. Automatic processing of rotation diffraction data from crystals of initially unknown symmetry and cell constants. *J. Appl. Crystallogr*. **1993**, *26*, 795–800.
3. Sheldrick, G.M. Crystal Structure Refinement with SHELXL. *Acta Cryst*. **2015**, *C71*, 3–8.
4. Dolomanov, O.V.; Bourhis, L.J.; Gildea, R.J.; Howard, J.A.K.; Puschmann, H. OLEX2: a complete structure solution, refinement and analysis program. *J. Appl. Crystallogr*., **2009**, *42*, 339–341.
5. Bryce-Smith, D.; Evans, E.H.; Gilbert, A.; McNeill, H.S. Photoaddition of ethenes to 1,4-naphthoquinone: factors influencing the site of reaction. *J. Chem. Soc. Perkin Trans.1* **1992**, 485–489.
6. Yaseen, M.A.; Mumtaz, S.; Hunter, R.L.; Wall, D.; Belluau, V.; Robertson, M.J.; Oelgemöller, M. Continuous-Flow Photochemical Transformations of 1,4-Naphthoquinones and Phthalimides in a Concentrating Solar Trough Reactor. *Austr. J. Chem.* **2020**, *73*, 1149–1157.
7. Maruyama, K.; Otsuki, T.; Takuwa, A.; Kako, S. Photochemical reaction of 1, 4-Naphthoquinone with olefins. *Bull. Inst. Chem. Res., Kyoto Uni.* **1972**, *50*, 344–347.
8. Pappas, S.; Portnoy, N.A. Substituent effects on the photoaddition of diphenylacetylene to 1, 4-naphthoquinones. *J. Org. Chem.* **1968**, *33*, 2200–2203.
9. Farid, S.; Kothe, W.; Pfundt, G. NMR-study of cyclobutene derivatives. *Tetrahedron Lett.* **1968**, *9*, 4151–4154.
10. Chen, X.; Huang, C.; Zhang, W.; Wu, Y.; Chen, X.; Zhang, C.-Y.; Zhang, Y. A universal activator of micro RNAs identified from photoreaction products. *Chem. Comm.* **2012**, *48*, 6432–6434.
